# Study on progressive failure mode of surrounding rock of shallow buried bias tunnel considering strain-softening characteristics

**DOI:** 10.1038/s41598-024-60324-y

**Published:** 2024-04-26

**Authors:** Xiaoxu Tian, Zhanping Song, Xiaole Shen, Qinsong Xue

**Affiliations:** 1 School of Civil Engineering, Xi’an University of Architecture and Technology, Xi’an, 710055 China; 2 Shaanxi Key Lab of Geotechnical and Underground Space Engineering, Xi’an, 710055 China; 3 No. 1 Engineering Corporation Limited of CR20G, Suzhou, 215151 China

**Keywords:** Shallow buried bias tunnel, Strain softening, Elastic–plastic analysis, Progressive failure mode, Civil engineering, Mechanical engineering, Natural hazards

## Abstract

Mountain tunnels portal often have to pass through slope terrain unavoidably, thus forming a shallow buried bias tunnel. During the construction of shallow buried bias tunnel, disasters such as slope sliding and tunnel collapse frequently occur. The failure mode of surrounding rock obtained by current research is based on the limit equilibrium theory, which cannot reflect the progressive failure characteristics of the surrounding rock of shallow buried bias tunnel. In order to reveal the failure mechanism of the gradual instability of surrounding rock of shallow buried bias tunnel, the problem of gradual failure of the surrounding rock is reduced to an elastic–plastic analysis problem for surrounding rock considering the strain-softening characteristics. Based on the elastic–plastic analysis of the failure process of shallow buried bias tunnel, MATLAB was used to compile a program to read the finite-difference calculation result file, extract the effective information such as shear strain and tensile strain at the center point of each unit, and establish the analysis method of the progressive failure mode of shallow buried bias tunnel. The reliability of the method proposed was verified by comparing the failure process of the model test with the development process of shear strain increment. Under the condition of no support, the formation mechanism of failure plane of surrounding rock on both sides of shallow buried bias tunnel is different. The shallow buried side is the shear failure plane formed by the collapse of surrounding rock, while the deep buried side of the tunnel is the shear failure plane formed by the collapse of surrounding rock and slope sliding. Under the conditions of excavation and support, the failure plane of the shallow buried bias tunnel can be divided into three parts according to the formation sequence and reasons. The part I is the failure plane, which is formed by active shear under the influence of tunnel excavation. The part II is the failure plane formed by tensile crack of slope top. The part III is the failure plane formed by passive shear under the push of the soil in the upper part of the slope.

## Introduction

The terrain of mountainous area is undulating, and the portal of many tunnels is bound to be located in the slope, forming shallow buried bias tunnel^1,2^. A large number of engineering cases show that many shallow buried bias tunnels have large deformation, cracking and slope sliding disasters after the first lining or the liner construction for a period of time^3^. The investigation found that the main cause of these disasters is that the actual load of the tunnel structure is not consistent with the design surrounding rock pressure in size and direction^4^. However, the surrounding rock failure pattern is the key factor affecting the size and direction of the surrounding rock pressure. It is necessary to further study the surrounding rock failure pattern of the shallow buried bias tunnel.

For the shallow buried bias tunnel, Minardo et al.^5^ pointed out that the deformation caused by unloading during the excavation of eccentrically pressurized zone of the tunnel portal will react on the tunnel structure, causing it to be in an unfavorable stress state. Therefore, the design and construction of the eccentrically pressurized zone of the tunnel portal should be targeted. Wang et al.^6^ discussed the failure mechanisms of landslides owing to tunnel excavation based on a three-dimensional numerical simulation, and evaluated the effects of proposed reinforcement measures. Zhang et al.^7^ proposed a pile-anchor support system for the tunnel in landslide. The sliding force is resisted by anti-sliding piles, while the tunnel deformation is limited by anchor cables buried in the opposite direction of the landslide. The superiority of the pile-anchor support system was verified by model tests. Wu et al.^8^ put forward a two-layer first lining for deep-buried tunnels with uneven load, which effectively controlled large deformation of surrounding rock. Combined with the results of field tests and field investigations, anti-slide piles + backfilling + slope brushing protective measures were proposed to strengthen the stability of the shallow buried bias tunnel^9^. Ayoublou et al.^10^ analyzed and summarized the deformation and stress characteristics of the tunnel portal section, and gave the construction suggestions. Sun and Fan et al. studied the disaster mechanism of the loess tunnel portal through numerical simulation and model test, and put forward deformation control measures for tunnel excavation^11,12^.

The primary cause of slope sliding and tunnel collapse during tunnel excavation often stems from an inadequate understanding of the mechanical characteristics^13,14^. A comprehensive comprehension of the deformation characteristics of the lining is a essential prerequisite for tunnel design and addressing potential disaster of shallow buried bias tunnel. Therefore, Liu et al.^15^ proposed an upper bound solution for assessing the surrounding rock pressure in shallow buried bias tunnel by using the principles of virtual work and the theoretical foundations of the limit upper bound method. Wang^16^, through extensive monitoring of shallow buried bias tunnel, obtained the deformation and crack distribution characteristics of the tunnel lining and proposed a novel approach to identify the possible causes of lining deformation and cracking. Over the course of a 5-year monitoring endeavor, Poisel et al.^17^ diligently tracked and observed a shallow buried bias tunnel. They observed that the width of lining cracks increased at an annual rate of 1.5 mm, while the convergence rate escalated at a rate of 3.5 mm/year. This observation strongly suggests that the deformation and cracking of tunnel linings within slope exhibit significant time-dependent behavior. Chiu et al.^18^ confirmed this view based on field monitoring data. Ruggeri et al.^19^ and Wei et al.^20^, drawing on their analyses of tunnel and slope deformation monitoring data, discerned the substantial influence of rainfall on time-dependent deformation. This underscores the critical role of environmental factors, particularly rainfall, in shaping the behavior of shallow buried bias tunnel. Based on model tests, Lei et al.^21^ found that the whole process of the instability of shallow buried bias tunnel in homogeneous layer can be described as follows: tunnel excavation causes collapse deformation, then induces surface tension on the deep buried side of the tunnel, and finally causes deep shear of the surrounding rock on the deep buried side to form a failure surface. It proved that the instability process of shallow buried bias tunnel is a gradual cumulative failure process from local to whole.

To sum up, many scholars have established new failure modes based on the results of practical engineering and model tests. However, the above failure modes of surrounding rock are based on the limit equilibrium theory, which cannot reflect the progressive failure characteristics of the surrounding rock of shallow buried bias tunnel. At present, the research on the progressive failure process of surrounding rock is mainly focused on the qualitative description of laboratory tests and numerical simulation, and the progressive failure mode and analysis method of surrounding rock in shallow buried bias tunnel has not been proposed. Therefore, in this paper, the progressive failure problem of surrounding rock was reduced to an elastic–plastic analysis problem of surrounding rock considering strain softening constitutive, and an analysis method of progressive failure mode of surrounding rock was established to quantitatively study the progressive failure mode of surrounding rock in shallow buried bias tunnel.

## Progressive failure principle

According to the model test results of Lei^21^ et al., the instability failure of the surrounding rock of shallow buried bias tunnel in homogeneous layer is essentially a progressive failure process from local to whole. The redistribution of stress in surrounding rock caused by tunnel excavation causes the point of maximum shear stress in surrounding rock to exceed the peak shear strength and soften, and the shear strength of rock and soil body decrease after softening, so that the original shear stress of rock and soil body exceed its shear strength. The residual shear stress of the part exceeding the shear strength will be transferred to the adjacent unsoftened rock and soil mass, so that the shear stress of the adjacent part of the rock and soil mass will increase and exceed its peak shear strength, and then the strength will decrease after softening, and so on until the tunnel collapse or the slope slides. Therefore, the problem of progressive failure of surrounding rock in shallow buried bias tunnel can be attributed to the problem of elastic–plastic analysis of surrounding rock considering strain softening constitutive.

**Figure 1:**
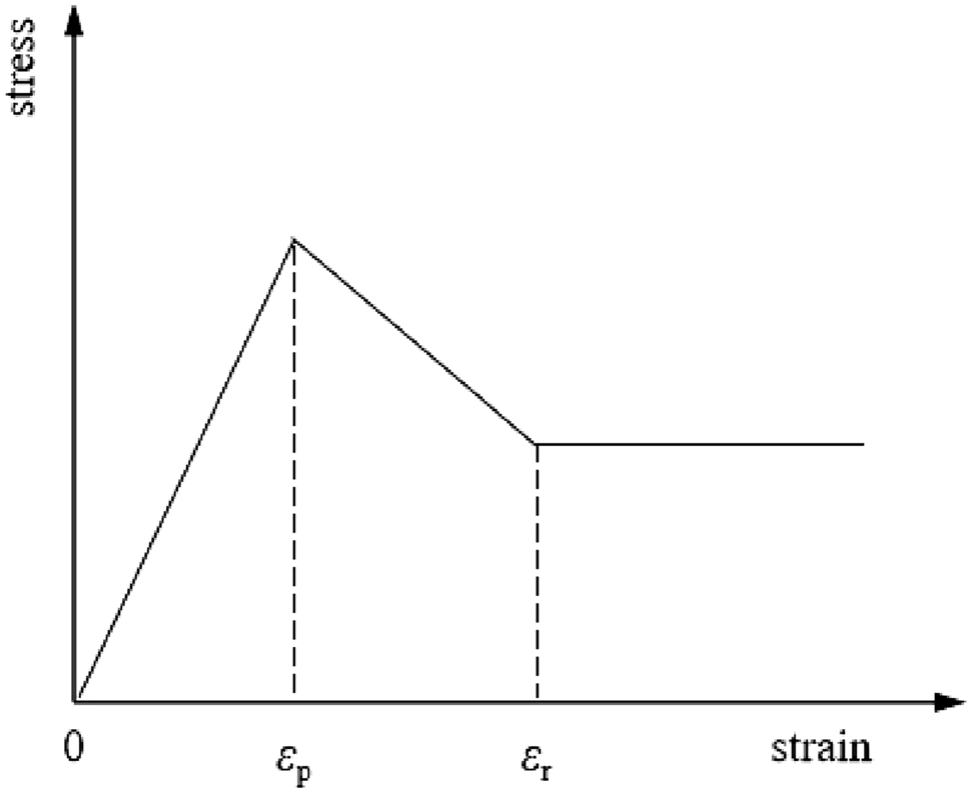
Linear softening model.

**Figure 2:**
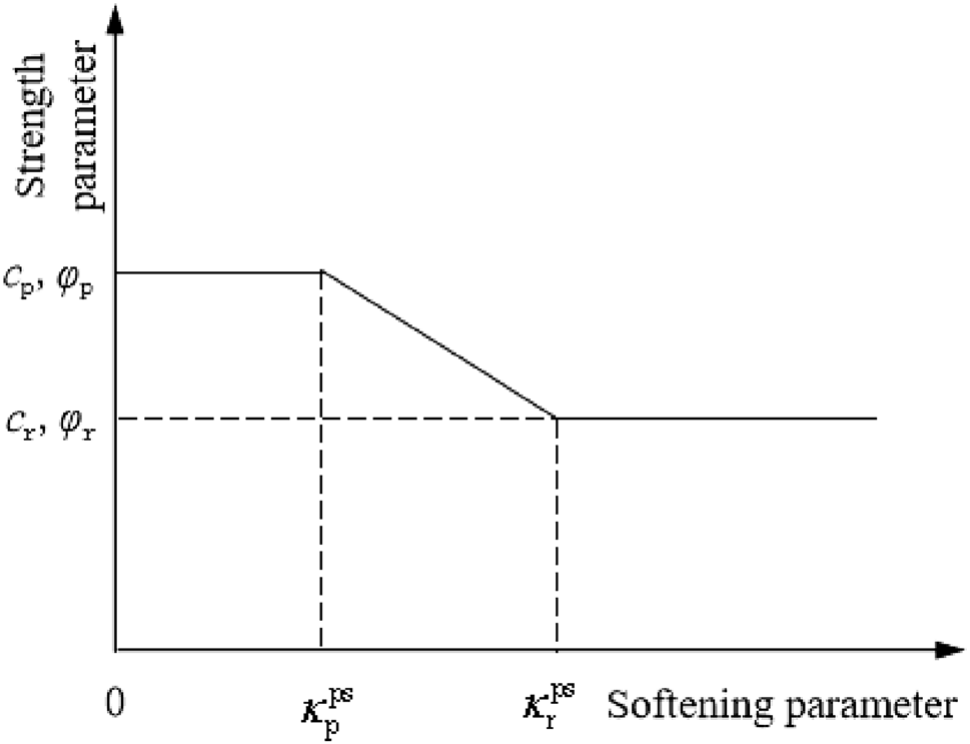
Linear strain -softening model parameters.

At present, there are many strain softening models, among which the linear strain softening model based on Mohr–Coulomb strength criterion has the advantages of simple, easy to obtain parameters and stable calculation, which is widely used in the analysis of progressive failure of surrounding rock and slope^22^. Therefore, the linear strain softening model based on Mohr–Coulomb strength criterion, and the stress–strain relationship is shown in Fig. 1. The selection of shear strength parameters is shown in Fig. 2. The specific expressions are shown in Eqs. (1)–(2); the expression of the strength criterion is shown in Eq. (3).

1 $$c = \left( {\begin{array}{*{20}l} {c_{p} } \hfill & {\kappa^{ps} \le \kappa_{p}^{ps} } \hfill \\ {c_{p} - \frac{{\kappa_{p}^{ps} - \kappa^{ps} }}{{\kappa_{p}^{ps} - \kappa_{r}^{ps} }}\left( {c_{p} - c_{r} } \right)} \hfill & {\kappa_{p}^{ps} < \kappa^{ps} < \kappa_{r}^{ps} } \hfill \\ {c_{r} } \hfill & {\kappa_{r}^{ps} \le \kappa^{ps} } \hfill \\ \end{array} } \right.$$

2 $$\varphi = \left( {\begin{array}{*{20}l} \varphi \hfill & {\kappa^{ps} \le \kappa_{p}^{ps} } \hfill \\ {\varphi_{p} - \frac{{\kappa_{p}^{ps} - \kappa^{ps} }}{{\kappa_{p}^{ps} - \kappa_{r}^{ps} }}\left( {\varphi_{p} - \varphi_{r} } \right)} \hfill & {\kappa_{p}^{ps} < \kappa^{ps} < \kappa_{r}^{ps} } \hfill \\ {\varphi_{r} } \hfill & {\kappa_{r}^{ps} \le \kappa^{ps} } \hfill \\ \end{array} } \right.$$

3 $$F\left( {\sigma ,\kappa^{ps} } \right) = \sigma_{1} - \frac{{1 + \sin \varphi \left( {\kappa^{ps} } \right)}}{{1 - \sin \varphi \left( {\kappa^{ps} } \right)}}\sigma_{3} + \frac{{2c\left( {\kappa^{ps} } \right)\cos \varphi \left( {\kappa^{ps} } \right)}}{{1 - \sin \varphi \left( {\kappa^{ps} } \right)}} = 0$$

where the incremental form of the shear-softening parameter is defined as $\vartriangle \kappa^{ps} = \sqrt {\left( {\vartriangle \varepsilon_{1}^{ps} - \vartriangle \varepsilon_{m}^{ps} } \right)^{2} + \left( {\vartriangle \varepsilon_{m}^{ps} } \right)^{2} + \left( {\vartriangle \varepsilon_{3}^{ps} - \vartriangle \varepsilon_{m}^{ps} } \right)^{2} } /\sqrt 2$, $\vartriangle \varepsilon_{1}^{ps}$ and $\vartriangle \varepsilon_{1}^{ps}$ correspond to the increment of plastic principal strain in the direction of principal stress $\sigma_{1}$ and $\sigma_{3}$, respectively. $\sigma_{1}$, $\sigma_{2}$ and $\sigma_{3}$, are the principal stresses respectively. $\sigma_{1} \le \sigma_{2} \le \sigma_{3}$, $\vartriangle \varepsilon_{m}^{ps}$ corresponds to the strain increment of the plastic shear body, $\vartriangle \varepsilon_{m}^{ps} { = }\left( {\vartriangle \varepsilon_{1}^{ps} { + }\vartriangle \varepsilon_{3}^{ps} } \right)/3$, $c_{p}$ and $\phi_{p}$ are the peak intensity parameters, $c_{r}$ and $\phi_{r}$ are residual strength parameters, $\kappa_{p}^{ps}$ and $\kappa_{r}^{ps}$ are the thresholds of plastic shear strain corresponding to the peak time and residual time respectively.

## Implementation and verification

The progressive failure characteristics of the surrounding rock of shallow buried bias tunnel can be analyzed by the strain softening characteristics of the material. But to study the failure mode of the surrounding rock, the distribution form and location of the failure surface of the surrounding rock must be determined first.

### Determination method of failure surface of surrounding rock

According to Mohr–Coulomb strength theory, the failure of rock and soil mass must be caused by the shear stress on one side reaching the shear strength of rock and soil mass. At this time, large shear deformation must occur on the shear surface. Therefore, the position of maximum shear strain increment can be used to determine the slope sliding surface search^23,24^. Based on this, the slope sliding surface determination method based on the maximum shear strain search is introduced into the study of the tunnel surrounding rock, and the method of determining the surrounding rock failure surface based on the maximum shear strain increment is proposed.

According to the study of Sun et al.^25^, a series of vertical lines can be set in the horizontal direction inside the shallow buried bias tunnel model during the elastic–plastic analysis of surrounding rock, and it is considered that the position of maximum shear strain on the vertical line is the position where the failure surface passes through. Because all the stress and strain data of the unit calculated by FLAC are the data of the central point of the unit, the failure surface pattern obtained is often not smooth and has a fluctuating pattern, which is inconsistent with the reality. Therefore, the density of the vertical line setting is as high as possible, and it also needs to be smooth treatment.

In the homogeneous layer, it is assumed that the vertical and lateral coordinates of the maximum point of equivalent plastic strain are *y*_*j*_ and *x*_*j*_, 1, 2, *j*… *n*, the functional form of the failure surface can be expressed as:

4 $$X\left( x \right) = \sum\limits_{k}^{K} {c_{k} } \phi_{k} \left( x \right) = {\mathbf{c}}^{{\mathbf{T}}} {\mathbf{\varphi }}$$

where ϕ_*k*_(*x*) is the basis function; *c*_*k*_ is the corresponding coefficient; ***φ*** and **c**^**T**^ are matrix forms. To define an *n* × *k* matrix **ψ** containing the data *ϕ*_*k*_(*x*_*j*_), the following minimum objective function is required^24^ :

5 $$F\left( {y\left| c \right.} \right) = \sum\limits_{k}^{K} {\left[ {y_{j} - \sum\limits_{k}^{K} {c_{k} \phi_{k} \left( {x_{j} } \right)} } \right]}^{2}$$

The matrix form is:

6 $$F\left( {y\left| c \right.} \right) = \left( {{\mathbf{Y - \psi c}}} \right)^{{\mathbf{T}}} \left( {{\mathbf{Y - \psi c}}} \right) = \left\| {{\mathbf{Y - \psi }}\left. {\mathbf{c}} \right\|} \right.^{2}$$

where **Y** is the column vectors of *y*_*j*_ .

Formula (6) differential is:

7 $$2{{\varvec{\uppsi}}}^{{\mathbf{T}}} {\mathbf{\psi c}} - {\mathbf{2\psi }}^{{\mathbf{T}}} {\mathbf{Y}} = 0$$

By solving this equation formula (7), we can obtain the value of vector **c** when the objective function takes the minimum value:

8 $${\mathbf{c}} = \left( {{{\varvec{\uppsi}}}^{{\mathbf{T}}} {{\varvec{\uppsi}}}} \right)^{{{\mathbf{ - 1}}}} {{\varvec{\uppsi}}}^{{\mathbf{T}}} {\mathbf{Y}}$$

This gets the form of curve X(*x*) of the sliding surface.

When there are different soil layers in the surrounding rock, because the physical and mechanical parameters of each soil layer are different, the stress of the soil will be discontinuous at the junction of the soil layer, and the position function of the maximum equal effect variation of the search is fit by the soil layer^24^, Taking two layers of soil as an example, and there are:

9 $$\left. {\begin{array}{*{20}l} {2{{\varvec{\uppsi}}}_{1}^{{\mathbf{T}}} {{\varvec{\uppsi}}}_{1} {\mathbf{c}}_{1} - {\mathbf{2\psi }}_{1}^{{\mathbf{T}}} {\mathbf{Y}}_{1} = 0} \hfill \\ {2{{\varvec{\uppsi}}}_{2}^{{\mathbf{T}}} {{\varvec{\uppsi}}}_{2} {\mathbf{c}}_{2} - {\mathbf{2\psi }}_{2}^{{\mathbf{T}}} {\mathbf{Y}}_{2} = 0} \hfill \\ \end{array} } \right\}$$

where **ψ**_1_ and **Y**_1_ corresponding to the maximum point of the plastic strain within the soil layer 1. At the same time, the continuity of the failure surface at the soil layer junction is considered, and the two functions are continuous at the junction, there are:

10 $${\mathbf{c}}_{{\mathbf{1}}}^{{\mathbf{T}}} \phi \left( {x_{0} } \right) = {\mathbf{c}}_{{\mathbf{2}}}^{{\mathbf{T}}} \phi \left( {x_{0} } \right)$$

Joint Eqs. (9) and (10) with *n* + 1 equations, but only *n* variables:

11 $${\mathbf{Ac}} = {{\varvec{\uprho}}}$$

among,

12 $$A = \left[ {\begin{array}{*{20}l} {{{\varvec{\uppsi}}}_{1}^{{\mathbf{T}}} {{\varvec{\uppsi}}}_{1} } \hfill & 0 \hfill \\ 0 \hfill & {{{\varvec{\uppsi}}}_{2}^{{\mathbf{T}}} {{\varvec{\uppsi}}}_{2} } \hfill \\ {\phi^{{\mathbf{T}}} \left( {x_{0} } \right)} \hfill & { - \phi^{{\mathbf{T}}} \left( {x_{0} } \right)} \hfill \\ \end{array} } \right]$$

13 $${{\varvec{\uprho}}}{ = }\left[ \begin{gathered} {{\varvec{\uppsi}}}_{1}^{{\mathbf{T}}} {\mathbf{Y}}_{1} \hfill \\ {{\varvec{\uppsi}}}_{2}^{{\mathbf{T}}} {\mathbf{Y}}_{2} \hfill \\ 0 \hfill \\ \end{gathered} \right]$$

The least squares solution of Eq. (11) is taken as the parameter vector **c** to define the sliding surface. For the surrounding rock with multiple soil layers, according to the specific situation and continuity condition of Eq. (10), whether to add the piecewise function equations required to solve. If the physical and mechanical properties of each soil layer are very different or there is a weak interlayer, there is a fault between the two sections of failure surface at the junction of the soil layer, and a section of failure surface in the junction is in line with the actual situation, there is no need to add. If the mechanical properties of each soil layer are not very different, they can be considered to ensure the continuity of the whole function^24^.

### Determination process of failure surface of surrounding rock

**Figure 3:**
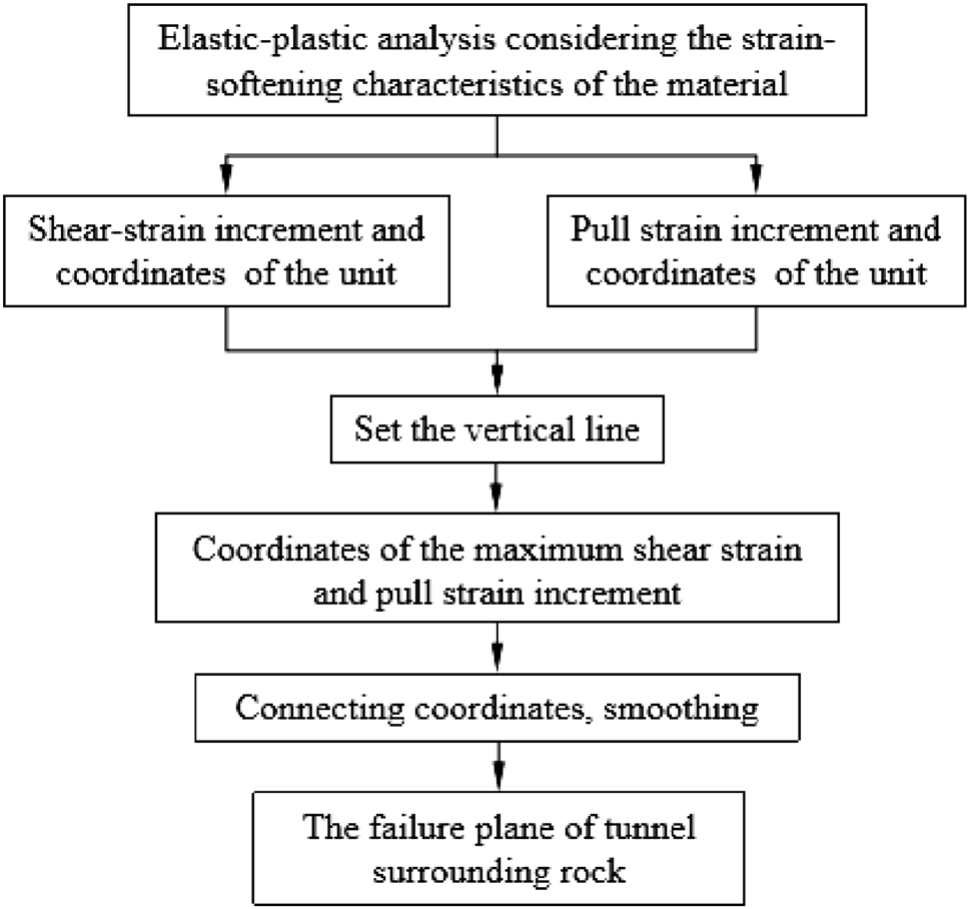
Determination process of surrounding rock failure plane.

**Figure 4:**
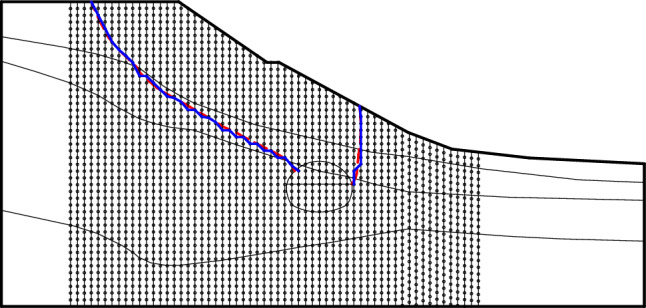
Schematic diagram of search method for surrounding rock failure plane based on maximum shear strain increment.

The implementation process of the progressive failure mode of the surrounding rock of the shallow buried bias tunnel is shown in Fig. 3. (i) elastic–plastic analysis of the shallow buried bias tunnel surrounding rock for stress strain field; (ii) The program is programmed with Matlab to read the finite-difference calculation result file and extract the effective information such as shear strain and tensile strain at the center point of each unit; (iii) a series of vertical lines are set in the horizontal direction inside the slope body, as shown in Fig. 4.

The initial failure surface can be obtained by searching the coordinates of points with the largest shear strain increment on each vertical line and connecting them. Then the initial failure surface is smoothened by the least square method, and the final failure surface position can be obtained.

### Verification of progressive failure mode analysis method of surrounding rock

The model test of a tunnel excavation free collapse^21^ is taken as an example. The finite difference software FALC is used to simulate the progressive failure process of the surrounding rock of shallow buried bias tunnel considering the strain softening characteristics. The analysis results are compared with model tests to verify the reliability of the failure mode analysis method for shallow buried bias tunnel surrounding rock considering progressive failure characteristics.

**Figure 5:**
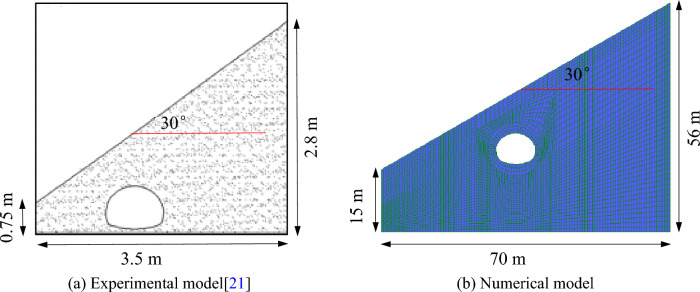
Experimental model and numerical model.

The model uses a mixture of gypsum and clay, slag and river sand as similar materials for lining and surrounding rock. The final ratio of material is: for lining, the mass ratio of gypsum and water is 1:0.75; for surrounding rock, the mass ratio of clay, slag and river sand is 1:1:2. The physical and mechanical parameters of the materials are shown in Table 1. The geometric similarity ratio of the model is 1:20, and the size of the model box is 3.5 m × 3.0 m × 2.0 m (length, width and height). With the slope of 30°, the model test results and numerical analysis results of tunnel excavation free collapse are compared and analyzed. The model was divided into 82,626 units and 37,306 nodes. The model is shown in Fig. 5.

**Table 1:** Physical and mechanical parameters of materials^21^.

Name	E/kPa	γ/kN/m^3^	*μ*	Peak strength		Residual strength	
				c/kPa	φ/°	c/kPa	φ/°
Surrounding rock	1.0E + 06	19	0.45	30	18	25	15
Liner	2.8E + 07	25	0.2	–	–	–	–

**Figure 6:**
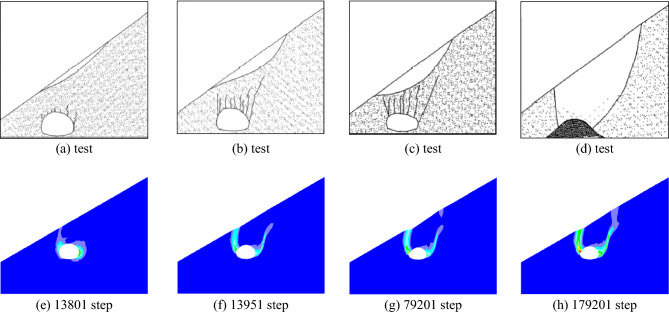
Comparison of model test and numerical test.

When the slope is 30°, the model test results of tunnel excavation free collapse failure process and the numerical analysis results of tunnel surrounding rock failure mode considering progressive failure characteristics are shown in Fig. 6. By comparing the model test results of different stages with those of numerical tests, it is found that the progressive development of plastic shear strain obtained by numerical simulation is consistent with the failure process of model tests. After tunnel excavation, the shallow buried side of the tunnel surrounding rock begins to form a shear failure zone from the position of the side wall and expands to the surface. The deep buried side surrounding rock forms a shear failure zone at the position of the arch foot and expands towards the top of the slope. The shear failure zone of shallow buried side first extends to the surface, and then the shear failure zone of deep buried side also extends to the surface, resulting in the overall collapse failure.

**Figure 7:**
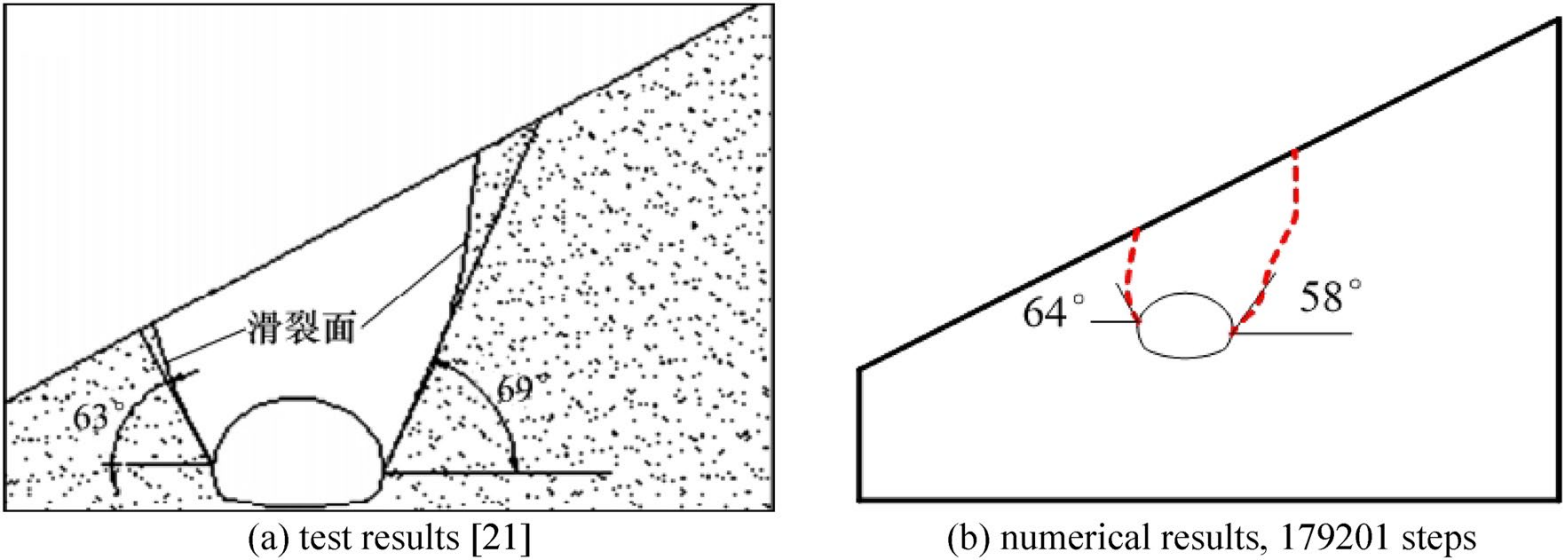
Failure plane of surrounding rock.

The failure plane formed by tunnel free collapse obtained by model test and numerical test is shown in Fig. 7, and the comparison results are shown in Table 2. The fracture starting position of the surrounding rock on both sides of the tunnel is the same as that obtained by the model test and the numerical test. The slope toe is at the side wall position and the slope top side is at the arch foot position. Although there are differences in values, the numerical test is highly consistent with the model test in terms of the failure mode of surrounding rock, which also shows that the analysis method of the failure mode of shallow buried bias tunnel surrounding rock considering the progressive failure characteristics has a certain reliability.

**Table 2:** Comparison of numerical results and experimental results of fracture angle.

Slope angle	Slope toe side		On one side of the top of the slope	
	Model test	Numerical test	Model test	Numerical test
30°	63°	64°	69°	58°

## Case study

**Figure 8:**
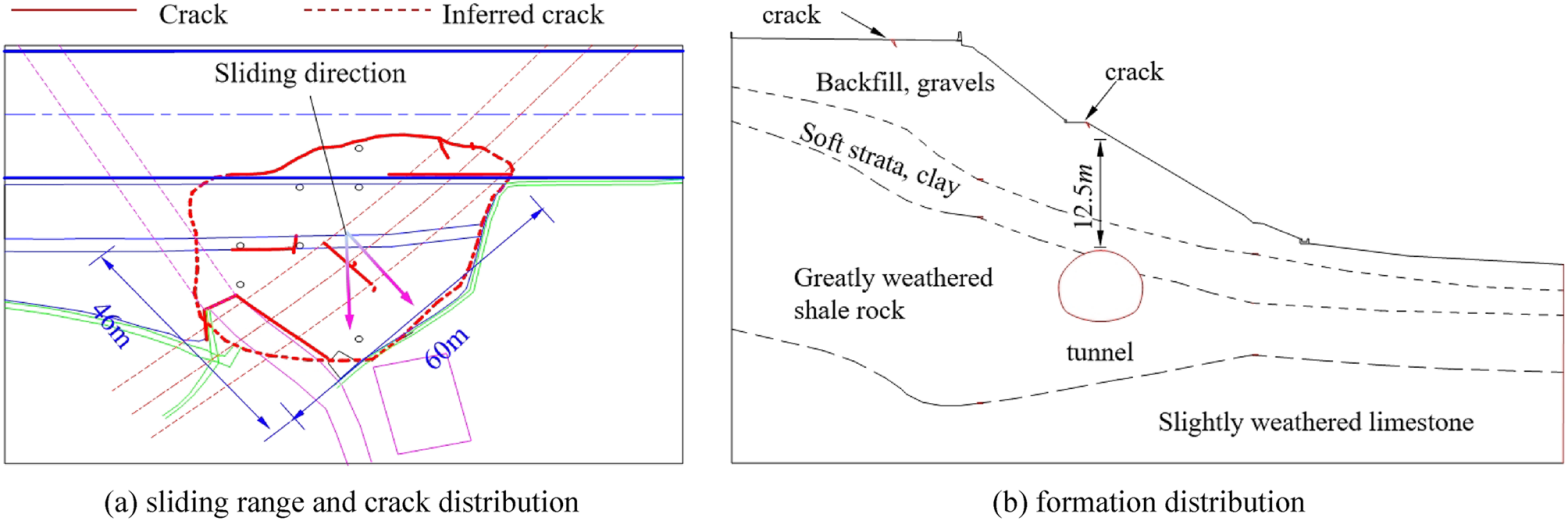
Slope slip range, fracture distribution and soil layer distribution.

In order to further verify the reliability of the progressive failure mode analysis method proposed in this paper, Xiamaixi tunnel is taken as an engineering example, and the numerical simulation results are compared with the actual monitoring data. The total length of the tunnel is 528 m. The tunnel is excavated by CRD method, the tunnel width is 11.8 m, the tunnel height is 8.7 m. The tunnel mostly passes through the limestone and shale strata, with a buried depth of 7.7 m-33 m, which is a typical shallow buried bias tunnel. The tunnel was constructed to YD1K1 + 456 on 15 August, 2013. It was suspended from August 15, 2013 to May 24, 2014 and resumed on May 25, 2014. On June 10, 2014, the tunnel face construction was YD1K1 + 467, the monitoring result of the slope displacement was abnormal, and the construction of the tunnel was suspended. According to the field investigation, there are several cracks in the top of the slope and the upper slope, and the landslide body is 60 m along the axis of the tunnel and 46 m along the transverse direction of the tunnel. The crack and soil layer distribution of the slope are shown in Fig. 8.

**Figure 9:**
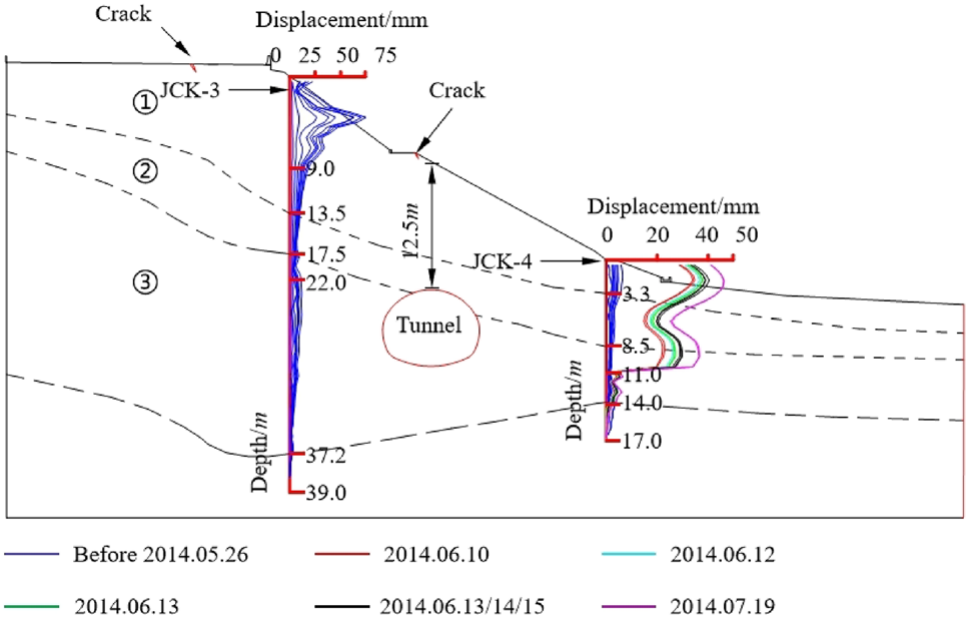
Slope horizontal displacement.

After the forced shutdown on August 15, 2013, in order to monitor the slope deformation, a total of two deep displacement monitoring points were installed on the slope, as shown in Fig. 9. From June 10, 2014 to July 19, 2014, the slope reinforcement construction damaged the monitoring point JCK-3, so there was no monitoring data at the monitoring point JCK-3 after June 10, 2014. During the tunnel shutdown, a large horizontal displacement occurred at monitoring point JCK-3, and little displacement occurred at monitoring site JCK-4. It shows that during the excavation to YD1K1 + 456, there is already a small sliding surface, but the sliding plane does not penetrate the whole slope. Therefore only the upper side of the slope body (the left side of the tunnel) has a large horizontal displacement. Before 26 May, 2014, the slope backfill layer had slipped. On June 10, 2014, the displacement of the monitoring point JCK-4 suddenly increased with an amplitude of 30 mm, which was consistent with the occurrence of large cracks at the site. The slip of the slope develops to the deeper soil layer, and the soft strata is deformed. It indicates that when the pipe-roof was constructed, and the tunnel excavation continued, the excavation unloading and disturbance caused the existing small sliding surface to pass through the complete slope, which leads to the complete slope sliding along the soft strata. The third layer at the slope toe was also deformed, which is consistent with the distribution of cracks in the drainage ditch at the slope toe.

**Figure 10:**
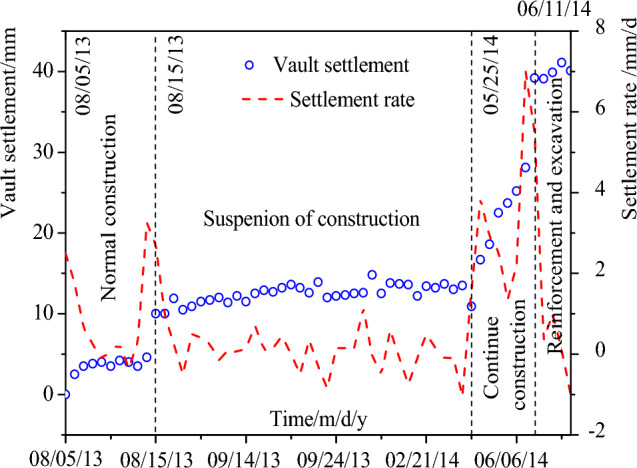
Settlement of the tunnel vault.

According to the the vault settlement monitored at YD1K1 + 450, the change curve of the vault settlement with time and the vault settlement rate with time are shown in Fig. 10. During normal construction, the maximum vault settlement is 10 mm, and the vault settlement is normal. During the suspension of construction, the vault settlement tends to be stable, but the settlement rate is not 0, and it is still increasing at a small rate, and the final settlement is 13.0 mm. During the tunnel reconstruction stage, the vault settlement sharply increases, the settlement rate increases, and the maximum vault settlement value is 28.1 mm. During the tunnel reinforcement and excavation, the settlement rate decreased, and the vault settlement value was 39.2 mm. As of 19 June, 2014, the vault settlement rate was close to 0.

The above monitoring data analysis shows that during the suspension of construction, the vault settlement increased by 3 mm, indicating that the deformation in the slope (namely the vault settlement) is small. The displacement of the two monitoring points (JCK-3 and JCK-4) of the slope is 60 mm and 5 mm respectively, indicating that the top of the slope is greatly deformed and the displacement of the slope toe is relatively small. This shows that after tunnel excavation, the stable state of the slope is changed, and the slope is still sliding along the soft strata at a small rate even if the construction of the tunnel does not continue. Then, when the slope protection is not well done, the tunnel continues to be excavated, which further reduces the anti-sliding force of the slope, resulting in the instability of the slope. This shows that the surrounding rock of shallow buried bias tunnel is a progressive instability process.

### Numerical model

The FLAC was used to simulate the progressive instability failure process, and the increment or plastic strain in the slope caused by tunnel excavation is analyzed, and the progressive instability failure mode of the surrounding rock of shallow buried bias tunnel is explored. The model uses the linear strain softening model based on the Mohr–Coulomb intensity criterion mentioned in Sect. “Progressive failure principle”.

**Figure 11:**
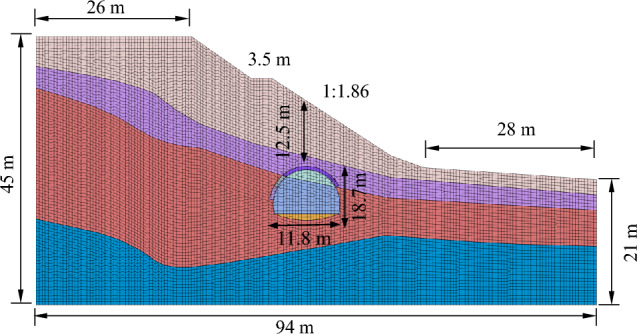
Numerical model.

The slope is 24 m high, the slope ratio is 1:1.86, the distance between the slope toe to the right boundary is equal to 28 m, the distance between the slope top to the left boundary is equal to 26 m, and the distance from the top to the bottom boundary of the slope is equal to 45 m. The model was divided into 12,626 units and 50,306 nodes. The upper surface of the model is free surface, horizontal displacement constraints are applied on the left and right sides, and full constraints are applied at the bottom of the model. The model is shown in Fig. 11, and the physical and mechanical parameters of the materials are shown in Table 3.

**Table 3:** Physical and mechanical parameters of materials.

Name	E/kPa	γ/kN/m^3^	*μ*	Peak strength		Residual strength	
				c/kPa	φ/°	c /kPa	φ/°
Backfill, gravels	4.00E + 05	20	0.43	20	30	15	18
Soft strata, clay	3.00E + 05	18	0.35	30	18	25	15
Greatly weathered shale rock	3.00E + 06	25.9	0.3	200	30	200	30
Slightly weathered limestone	3.50E + 06	26.4	0.3	450	35	450	35

### Failure mode of surrounding rock

**Figure 12:**
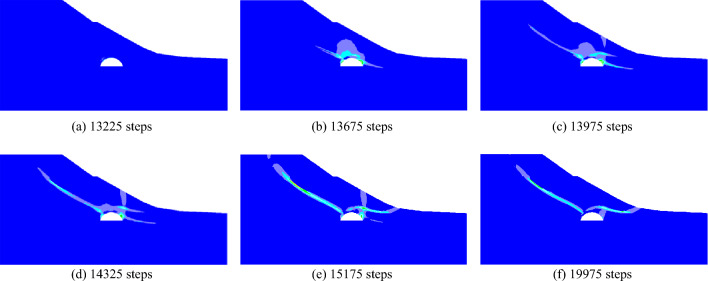
Contour of progressive development of plastic shear strain increment.

The progressive development process of plastic shear strain during the formation of failure plane of surrounding rock is shown in Fig. 12. After tunnel excavation, the shear failure area appears first in the tunnel vault area, and develops along the soft strata towards the slope top. There was no shear failure at the top and toe of the slope. With the increase of the calculation time step, the shear zone on the left side of the tunnel continues to expand upward along the soft strata. At the same time, the shear zone on the right side of the tunnel extends to the surface from the vault foot position and extends to the surface at step 14,325. However, no shear zone appears at the slope toe at this time. It shows that the surface settlement is large, the formation is mainly deformed into the tunnel, the slope is not completely formed, and the slope does not slip as a whole. With the formation deformation in the tunnel tends to be stable, the shear zone begins to expand to the slope toe. At step 15,175, the shear zone extends to the slope toe and shear failure occurs at the slope toe. At this time, the slope body is completely formed and the slope slips as a whole.

**Figure 13:**
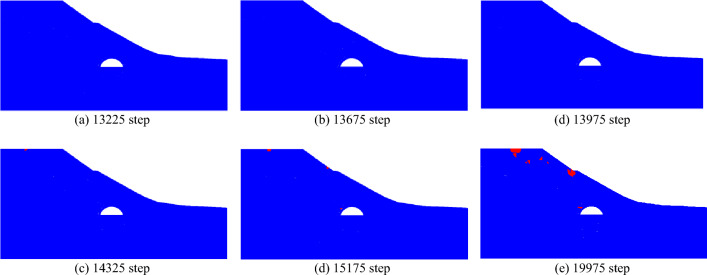
Contour of progressive development of plastic tensile strain increment.

The gradual development of plastic tensile strain in the slope is shown in Fig. 13. There is no tensile strain in the slope after tunneling. With the increase of the calculation step, the tensile strain appears at the top of 14,325 step, which just corresponds to the plastic shear strain on the tunnel right side extending to the surface, and the formation deforms into the tunnel. With the continuous increase of the calculation step, the tensile strain gradually appears in the upper part of the slope, which corresponds to the slope crack distribution investigated on site.

According to the above analysis, it can be seen that the first failure position of the slope is not the toe and the top of the slope, and its failure mode is neither traction landslide nor thrust-type landslide. The failure characteristics of the slope are as follows: the tunnel arch area is destroyed first, the shear zone on the tunnel left side develops along the soft strata to the slope top, and the shear zone on the tunnel right side extends from the arch foot through the soft strata to the surface. When the shear zone on the left is connected to the tensile zone on the slope top and the shear zone on the right extends to the surface, the surrounding rock displaces to the tunnel. When the displacement in the tunnel is close to stability, the soil of the slope upper part pushes the lower part soil along the soft strata to the slope toe, and the slope eventually fails as a whole.

**Figure 14:**
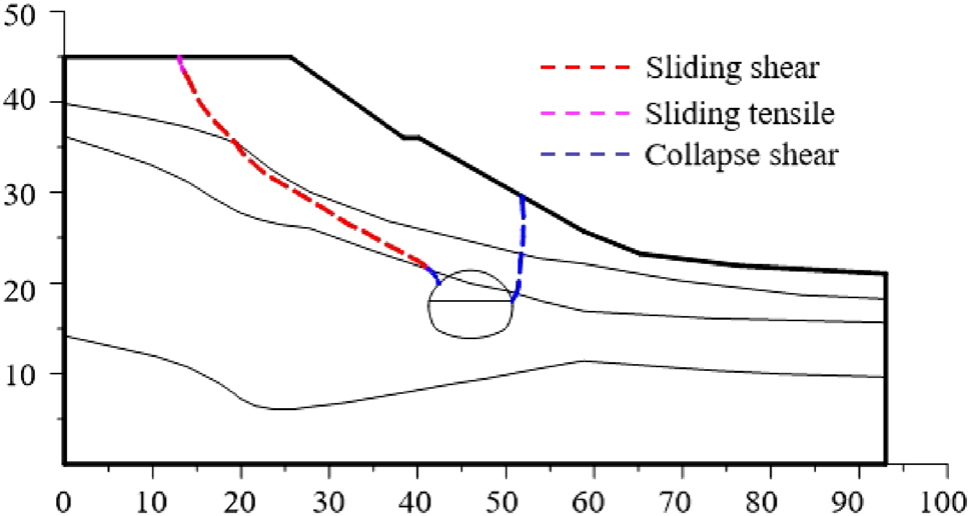
Failure plane of surrounding rock without support.

According to the process in Sect.  2.2, the program compiled by MATLAB is used to identify the points of maximum shear strain or maximum tensile strain increment on each vertical line, and then connect them to get the failure surface of surrounding rock after smooth processing. Before the slope sliding as a whole, the failure plane formed by the free collapse of the tunnel surrounding rock is shown in Fig. 14.

The failure plane of the shallow buried side of the tunnel extends from the arch foot to the surface. The failure plane not extends to the surface along the failure plane of the greatly weathered shale rock, but extends to the surface along the soft strata to the slope top. It shows that the failure plane exhibits sliding behavior rather than collapsing behavior.

**Figure 15:**
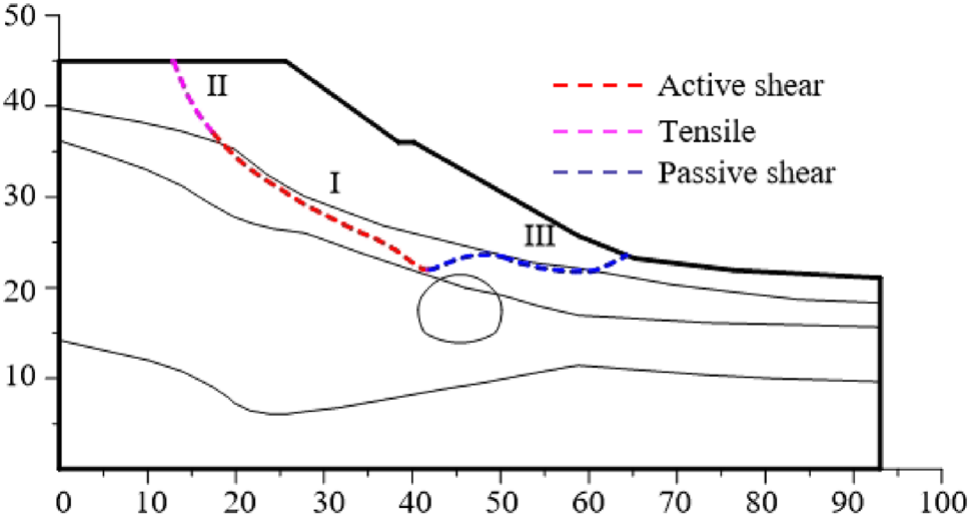
Slope sliding plane under excavation and support.

After the first lining is applied, the failure plane of the slope is shown in Fig. 15. The failure plane of the slope is divided into three parts according to the formation sequence and reasons.

The Part I is the first failure plane formed by active shear due to the influence of tunnel excavation; The part II is the failure plane formed by tensile of slope top; The part III is the failure plane formed by passive shear under the push of the soil in the upper part of the slope. According to the above progressive failure process of tunnel surrounding rock, it can be seen that the tunnel is first subjected to the collapse load of rock and soil mass in the process of excavation, and then subjected to the additional load caused by slope sliding.

## Analysis and discussion

The slope slipping and large deformation induced by tunnel excavation often occur in the shallow buried bias area of mountain tunnel portal because the covered soil is mostly accumulated body. Therefore, the progressive failure mode of the surrounding rock when the tunnel passes through the soil-rock interface is further analyzed. The progressive failure mode of the surrounding rock of shallow buried bias tunnel under uniform formation is compared and analyzed.

### Failure mode of surrounding rock of under tunnel passing through the soil-rock interface

**Figure 16:**
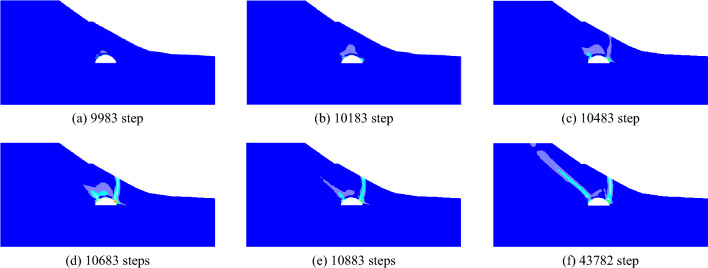
Contour of progressive development of plastic shear strain increment.

In order to investigate the progressive failure mode of the surrounding rock of shallow buried bias tunnel when there is soil-rock interface. Assuming that the first layer of surrounding rock of Xiamaixi Tunnel is backfill soil and the second layer is strongly weathered shale. The elastic–plastic analysis of surrounding rock is carried out under the same working condition. The progressive development process of plastic shear strain during the formation of failure plane in slope under the condition of tunnel excavation without support is shown in Fig. 16. After tunnel excavation, shear failure occurs first in the tunnel vault area. With the increase of the calculation time step, the shear zone of the shallow buried side of the tunnel, which starts from the arch foot, expands to the surface. The shear zone on the deep side of the tunnel extends along the combination of soil and rock towards the top of the slope, and finally extends to the surface.

**Figure 17:**
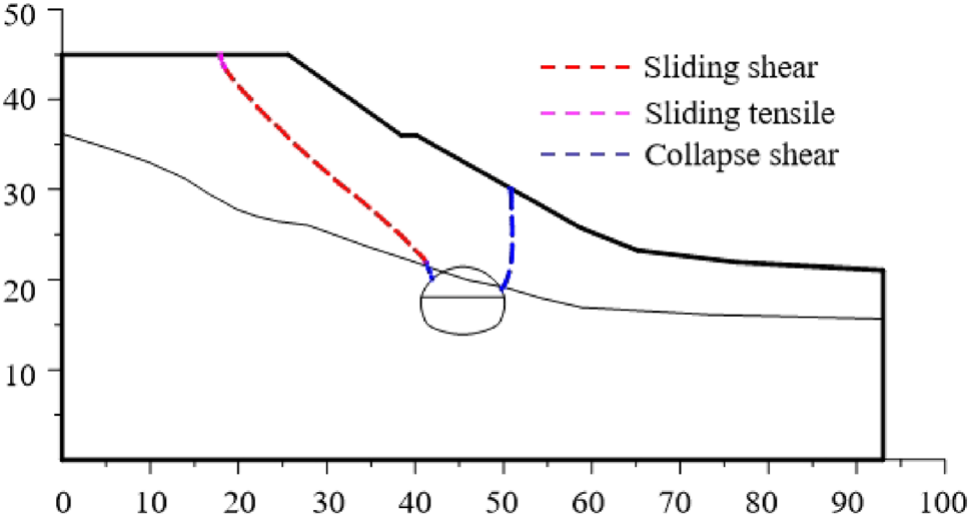
Failure plane of surrounding rock without support.

The final failure plane (potential failure plane) of surrounding rock is shown in Fig. 17. Similar to the failure plane formed by tunnel excavation without support when there is soft strata in surrounding rock, the failure plane of shallow buried side shows collapse characteristics, while the deep buried side mainly shows sliding characteristics. The failure plane of the shallow buried side shows collapsing behavior, while the deep buried side mainly shows sliding behavior.

**Figure 18:**
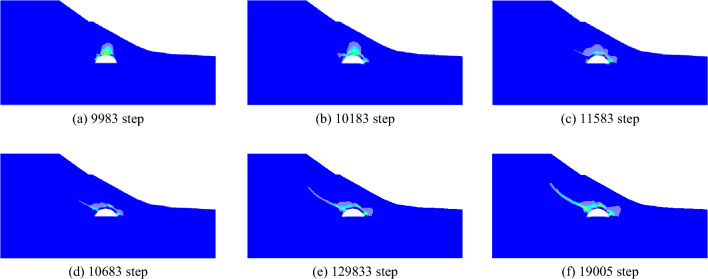
Contour of progressive development of plastic shear strain increment.

The development process of plastic shear strain during the formation of failure plane in slope under tunnel with support is shown in Fig. 18. After tunnel excavation, shear failure occurs first in the tunnel vault area, and the shear failure zone extends to the top and toe of the slope along the soil-rock interface.

**Figure 19:**
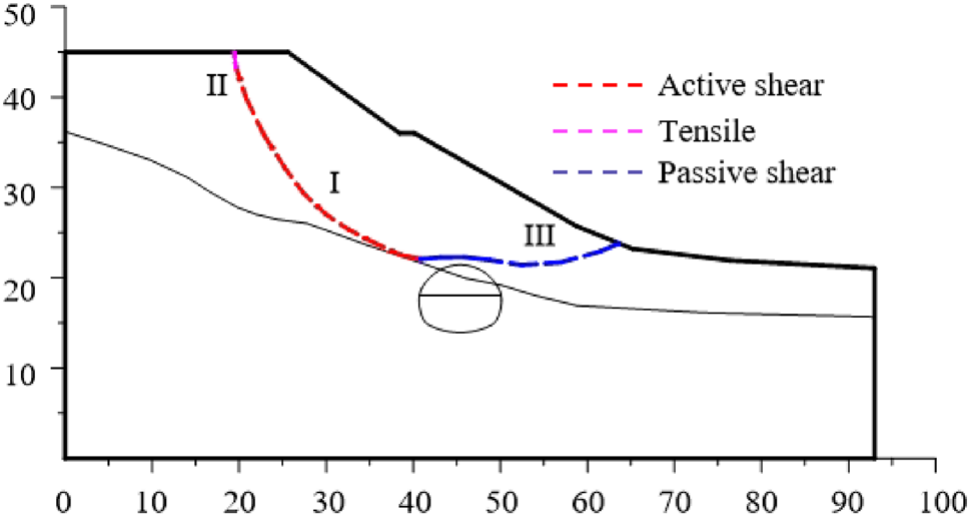
Slope sliding plane under excavation and support.

When the calculation time step is 19,005, the shear zone extends to the surface, forming a sliding plane (or potential sliding plane) as shown in Fig. 19. When the strength of tunnel support is enough to resist the collapse and deformation of surrounding rock, the collapse and deformation of surrounding rock gradually stabilizes. Therefore, the deformation of surrounding rock begins to be dominated by sliding deformation, and the slope eventually shows sliding deformation characteristics.

### Failure mode of surrounding rock in tunnel within homogeneous layer

**Figure 20:**
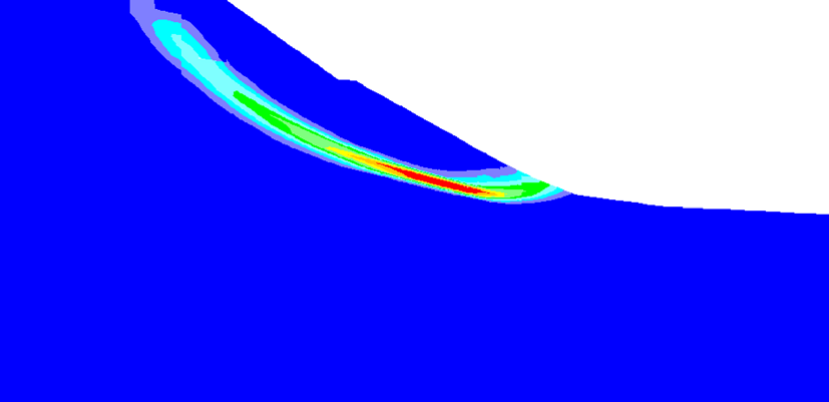
Contour of shear strain increment.

In order to explore the progressive failure mode of surrounding rock of shallow buried bias tunnel in homogeneous strata, assume that surrounding rock of Xiamaixi tunnel is greatly weathered shale, and the elastic–plastic analysis of surrounding rock is conducted under the same working condition. The strength reduction method was used to analyze the stability of the slope, and the calculated safety factor of the slope was 2.09, and the shear strain increment was shown in Fig. 20.

**Figure 21:**
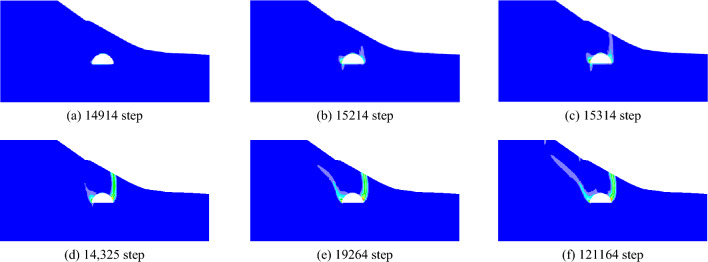
Contour of progressive development of plastic shear strain increment.

The gradual development of plastic shear strain in the formation of surrounding rock failure plane in the stable slope is shown in Fig. 21. After tunnel excavation, the shear zone appears in the arch foot area of the excavation section. With the increase of calculation time, the shear zone of the arch foot on both sides of the tunnel expands to the surface respectively. The shear zone of the arch foot on the shallow buried side of the tunnel has a faster expansion rate and first expands to the surface. Then the shear zone of the deep buried side arch foot also extends to the surface, thus forming the tunnel collapse failure plane and sliding failure plane.

**Figure 22:**
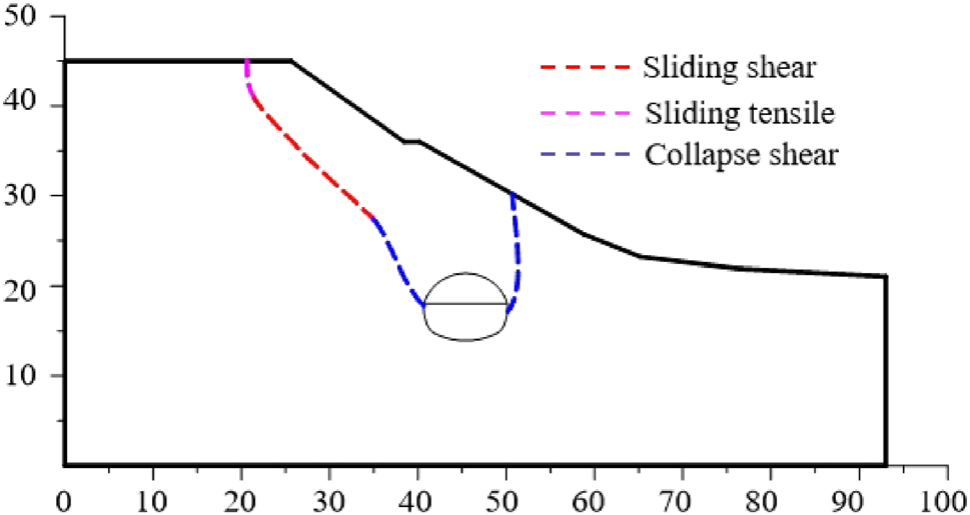
Failure plane of surrounding rock.

The failure plane or potential failure plane of surrounding rock is shown in Fig. 22. The failure plane of the deep buried side of the tunnel can be divided into three parts according to the causes. The first part is the failure plane formed by soil collapsing into the tunnel; The second part is the failure plane caused by the upper soil sliding along the slope; The third part is the failure plane caused by tensile of slope top.

Therefore, the deep buried surrounding rock exhibits collapse characteristics and sliding characteristics, the tunnel structure is actually subjected to both the collapse load of soil layer and the load generated by slope sliding.

## Conclusions

The material strain softening constitutive model was introduced into the analysis of surrounding rock failure mode, and the analysis method of progressive failure mode of shallow buried bias tunnel was established, and then the reliability of this method was verified according to the comparison of model test. Finally, this method is applied to the analysis of shallow buried bias tunnel to reveal the mechanism of progressive instability. The main results obtained are as follows:

1. The material strain softening constitutive model is introduced into the analysis of the tunnel surrounding rock failure mode. Based on the calculation results of the surrounding rock elastic–plastic analysis, the progressive failure mode of the surrounding rock of the shallow buried bias tunnel was analyzed by MATLAB software, and the reliability of the progressive failure mode analysis method of the surrounding rock of the shallow buried bias tunnel was verified.
2. Under the condition of no support, the formation mechanism of failure plane of surrounding rock on both sides of shallow buried bias tunnel is different. The shallow buried side is the shear failure plane induced by the collapse of surrounding rock, while the deep buried side of the tunnel is the shear failure plane induced by the collapse of surrounding rock and slope sliding.
3. The failure plane of the slope is divided into three parts according to the formation sequence and reasons. The Part I is the first failure plane formed by active shear due to the influence of tunnel excavation; The part II is the failure plane formed by tensile of slope top; The part III is the failure plane formed by passive shear under the push of the soil in the upper part of the slope. In the process of tunnel excavation, the tunnel is first subjected to the collapse load of rock and soil mass, and then subjected to the load caused by slope sliding. After the construction of the tunnel structure being completed, the load on the tunnel structure also includes the deformation effect from the slopes sliding, not just the soil collapse load.

## Data Availability

The datasets used and/or analysed during the current study are available from the corresponding author on reasonable request.

## References

[1] Fan SY, Song ZP, Xu T, Wang KM, Zhang YW (2021). Tunnel deformation and stress response under the bilateral foundation pit construction: A case study. Arch. Civ. Mech. Eng..

[2] Cheng Q, Xiao S, Liu T, Chen T, Li S, Wei A (2021). Twin tunneling–induced deep-seated landslide in layered sedimentary rocks. Bull. Eng. Geol. Environ..

[3] Chen H, Lai H, Huang M, Wang G, Tang Q, Glade T, Murty TS (2022). Failure mechanism and treatment measures of supporting structures at the portal for a shallow buried and asymmetrically loaded tunnel with small clear-distance. Nat. Hazards..

[4] Tian XX, Song ZP, Zhang YW (2021). Monitoring and reinforcement of landslide induced by tunnel excavation: A case study from Xiamaixi tunnel. Tunn. Undergr. Space Tech..

[5] Ma GT, Cao H, Tao ZG, Lu J, Zhu C (2023). Experimental study on deformation and failure characteristics and monitoring and early warning of surrounding rock of tunnel crossing sliding surface. Rock Mech. Rock Eng..

[6] Wang ZF, Shi FG, Li DD (2020). Tunneling-induced deep-seated landslides: A case study in Gulin county, Sichuan, China. Arab. J Geosci..

[7] Zhang ZG, Fang L, Zhao QH, Zhang M, Pan Y, Ma B (2022). An experimental evaluation of pile-anchor strengthening mechanics for existing tunnels in landslide region. Undergr. Space.

[8] Wu H, Fan FF, Yang XH, Lai J, Xie Y (2022). Large deformation characteristics and treatment effect for deep bias tunnel in broken phyllite: A case study. Eng. Fail. Anal..

[9] Zhang J, Kuang MX, Zhang YH, Feng T (2021). Evaluation and analysis of the causes of a landslide and treatment measures during the excavation of a tunnel through a soil-rock interface. Eng. Fail. Anal..

[10] Ayoublou FF, Taromi M, Eftekhari A (2019). Tunnel portal instability in landslide area and remedial solution: A case study. Acta Polytech..

[11] Sun ZJ, Yan XY, Lu S (2022). Influence of a landslide on a tunnel in loess-bedrock ground. Appl. Sci..

[12] Fan HB, Xu Q, Lai JX (2023). Stability of the loess tunnel foundation reinforced by jet grouting piles and the influence of reinforcement parameters. Transp. Geotech..

[13] Song ZP, Cheng Y, Zhang ZK, Yang TT (2023). Tunnelling performance prediction of cantilever boring machine in sedimentary hard-rock tunnel using deep belief network. J. Mt. Sci..

[14] Wu K, Song JA, Zhao NN, Shao ZS (2023). Study on the time-dependent interaction between surrounding rock and yielding supports in deep soft rock tunnels. Int. J. Numer. Anal. Met..

[15] Liu ZZ, Cao P, Lin H, Meng JJ, Wang YX (2020). Three-dimensional upper bound limit analysis of underground cavities using nonlinear Baker failure criterion. Trans. Nonferr. Metal Soc. China.

[16] Wang TT (2010). Characterizing crack patterns on tunnel linings associated with shear deformation induced by instability of neighboring slopes. Eng. Geol..

[17] Poisel R, Mairam Tinkhof K, Preh A (2015). Landslide caused damages in a gallery. Rock Mech. Rock Eng..

[18] Chiu YC, Lee CH, Wang TT (2017). Lining crack evolution of an operational tunnel influenced by slope instability. Tunn. Undergr. Space Tech..

[19] Ruggeri, P., Fruzzetti, V., Vita, A., Paternesi, A., Scarpelli, G. & Segato, D. Deep-seated landslide triggered by tunnel excavation. In: ISL 2016–12th International Symposium on Landslides. 2016;12–19. 10.1201/b21520-219.

[20] Wei H, Wu DY, Wu HG, Tang L, Wang S, Sun H (2023). Coordinated evolution and mechanism characteristics of the tunnel-landslide system under rainfall conditions. Eng. Fail. Anal..

[21] Lei MF, Peng LM, Shi CH (2015). Model test to investigate the failure mechanisms and lining stress characteristics of shallow buried tunnels under unsymmetrical loading. Tunn. Undergr. Space Tech..

[22] Xue HB, Dang FN, Yin XT, Lei M, Yang C (2016). Progressive failure characteristics of slopes considering strain-softening behavior of geotechnical materials and dynamics. Rock Soil Mech..

[23] Cheng YM, Liu HT, Wei WB, Au SK (2005). Location of critical three-dimension non-spherical failure surface by NURBS functions and ellipsoid with applications to highway slopes. Comput. Geotech..

[24] Cheng YM, Lansivaara T, Wei WB (2006). Two-dimensional slope stability analysis by limit equilibrium and strength reduction methods. Comput. Geotech..

[25] Sun GH, Zheng H, Li CG (2010). 3D slope surface search based on equivalent plastic strain. Rock Soil Mech..

